# Age-Related Difference in Functional Brain Connectivity of Mastication

**DOI:** 10.3389/fnagi.2017.00082

**Published:** 2017-04-03

**Authors:** Chia-shu Lin, Ching-yi Wu, Shih-yun Wu, Hsiao-Han Lin, Dong-hui Cheng, Wen-liang Lo

**Affiliations:** ^1^Department of Dentistry, School of Dentistry, National Yang-Ming UniversityTaipei, Taiwan; ^2^School of Dentistry, Institute of Oral Biology, National Yang-Ming UniversityTaipei, Taiwan; ^3^Division of Family Dentistry, Department of Stomatology, Taipei Veterans General HospitalTaipei, Taiwan; ^4^Division of Prosthodontics, Department of Stomatology, Taipei Veterans General HospitalTaipei, Taiwan; ^5^Division of Oral and Maxillofacial Surgery, Department of Stomatology, Taipei Veterans General HospitalTaipei, Taiwan

**Keywords:** aging, magnetic resonance imaging, mastication, functional connectivity, graph theory

## Abstract

The age-related decline in motor function is associated with changes in intrinsic brain signatures. Here, we investigated the functional connectivity (FC) associated with masticatory performance, a clinical index evaluating general masticatory function. Twenty-six older adults (OA) and 26 younger (YA) healthy adults were recruited and assessed using the masticatory performance index (MPI) and resting-state functional magnetic resonance imaging (rs-fMRI). We analyzed the rs-fMRI FC network related to mastication, which was constructed based on 12 bilateral mastication-related brain regions according to the literature. For the OA and the YA group, we identified the mastication-related hubs, i.e., the nodes for which the degree centrality (DC) was positively correlated with the MPI. For each pair of nodes, we identified the inter-nodal link for which the FC was positively correlated with the MPI. The network analysis revealed that, in the YA group, the FC between the sensorimotor cortex, the thalamus (THA) and the cerebellum was positively correlated with the MPI. Consistently, the cerebellum nodes were defined as the mastication-related hubs. In contrast, in the OA group, we found a sparser connection within the sensorimotor regions and cerebellum and a denser connection across distributed regions, including the FC between the superior parietal lobe (SPL), the anterior insula (aINS) and the dorsal anterior cingulate cortex (dACC). Compared to the YA group, the network of the OA group also comprised more mastication-related hubs, which were spatially distributed outside the sensorimotor regions, including the right SPL, the right aINS, and the bilateral dACC. In general, the findings supported the hypothesis that in OA, higher masticatory performance is associated with a widespread pattern of mastication-related hubs. Such a widespread engagement of multiple brain regions associated with the MPI may reflect an increased demand in sensorimotor integration, attentional control and monitoring for OA to maintain good mastication.

## Introduction

The traditional view holds that the individual differences in masticatory ability are predominantly predisposed by oral conditions, such as the number of teeth lost, the amount of biting force and the saliva flow rate (Hatch et al., 2001; Ikebe et al., 2011). However, as a highly coordinated rhythmic and automatic movement, mastication is also regulated by the central nervous system (CNS; Avivi-Arber et al., 2011). Neuroimaging evidence has suggested that mastication is associated with the brain activity in the cortical sensory and motor regions, the subcortical regions, and the cerebellum (Onozuka et al., 2002; Quintero et al., 2013). Furthermore, in elderly healthy subjects, good masticatory ability, as indexed by higher masticatory performance, was associated with greater gray matter volume at the premotor cortex (PMC) and an increased resting-state functional connectivity (FC) with the cerebellum (Lin et al., 2015). These findings highlighted that the individual differences in masticatory ability are not only associated with peripheral oral conditions but also the intrinsic brain signatures related to motor control.

The age-related difference in the CNS mechanisms of mastication has remained unclear. Neuroimaging findings have suggested that aging is associated with different patterns of brain activation for motor control (Mattay et al., 2002; Onozuka et al., 2003; Heuninckx et al., 2005; Wu and Hallett, 2005). First, when performing a simple motor task (e.g., finger tapping), older adults (OA) showed widespread activation in a distributed network, including in the sensorimotor area, the premotor area, the rostral cingulate the prefrontal cortex, the subcortical regions and the cerebellum, compared to that in younger adults (YA; Mattay et al., 2002; Heuninckx et al., 2005; Wu and Hallett, 2005). Such a widespread activation may indicate an age-related compensation in motor control or enhanced cognitive demand during movement (Ward, 2006; Seidler et al., 2010). Second, in OA, central pattern generator (CPG)-maintained movements (e.g., chewing, swallowing and locomotion) are engaged with extended cortical activation, including the insula, the prefrontal cortex and the parietal cortex (Onozuka et al., 2003; Zwergal et al., 2012; Windel et al., 2015; Lotze et al., 2016). Finally, evidence from resting-state FC showed a pronounced age-related change in the sensorimotor network (Tomasi and Volkow, 2012; Chan et al., 2014; Song et al., 2014; Fujiyama et al., 2016). The findings suggested that as age increases, a complex interplay between motor, sensory and cognitive processing, via altered FC, may play a predominant role in mastication.

The current study aimed to qualitatively and quantitatively compare the pattern of FC networks associated with masticatory performance between older and younger healthy adults. We adopted a graph-based method to construct the mastication-related network, which was composed of 12 bilateral brain regions associated with mastication. We identified the mastication-related “hubs”, which showed a positive correlation between masticatory performance and the degree centrality (DC) of the network. We hypothesized that, in older people vs. younger people, a higher masticatory performance would be associated with a widespread pattern of mastication-related hubs.

## Materials and Methods

### Study Group

Fifty-two healthy adults (33 female/19 male) aged 48.7 ± 18.0 (mean ± standard deviation) years old (y/o) were recruited. To investigate the age-related effect on masticatory performance and the related brain signatures, we median-split the participants into a subgroup of OA (>54.5 y/o, *n* = 26) and a subgroup of YA (<54.5 y/o, *n* = 26). Among the 52 participants, 21 were investigated in our previous study on the brain signatures associated with masticatory performance (Lin et al., 2015). The following exclusion criteria were applied: (1) having a history of major physical or psychiatric disorders, including epilepsy, major depression, schizophrenia or neurovascular diseases; (2) having a history of brain injury or receiving brain surgery; and (3) being unable to undergo MRI due to physical or psychological contraindications. The participants were recruited via advertisements in local community centers and the Taipei Veterans General Hospital. The study was approved by the Institutional Review Board of the National Yang Ming University and Taipei Veterans General Hospital, Taiwan. Written informed consent was provided by all participants before the experiment was initiated.

### Clinical and Neuroimaging Assessment

We adopted the colorimetric method based on color-changeable chewing gum (Hayakawa et al., 1998) for assessing individual masticatory function. After the MRI scan, each participant was asked to chew a piece of color-changeable chewing gum (Lotte Co. Ltd., Tokyo, Japan), which is designed for assessing masticatory performance (Hama et al., 2014), for 3 min. The chewed gum was flattened and scanned as a digitalized image (Schimmel et al., 2007), and the masticatory performance index (MPI) was quantified as the color change (ΔE) in the L*a*b* chromatic system (Hama et al., 2014) of the image.

### Acquisition and Preprocessing of Imaging Data

Resting-state functional MRI (rs-fMRI) was performed at the 3T MRI Laboratory of National Yang-Ming University using a 3 Tesla Siemens MRI scanner (Siemens Magnetom Tim Trio, Erlangen, Germany). The rs-fMRI images were acquired using a gradient echo EPI (Echo Planar Imaging) T2* weighted sequence ([TR] = 2000 ms, [TE] = 20 ms, matrix size = 64 × 64 × 40, voxel size = 3.4 × 3.4 × 3.4 mm^3^ and 183 volumes in total). During scanning, the participants were instructed to be relaxed but remain awake, keeping their eyes open and fixing on a cross symbol on the screen. Pre-processing of the rs-fMRI data was performed using the Data Processing Assistant for Resting-State fMRI (Chao-Gan and Yu-Feng, 2010) and the Resting-State fMRI Data Analysis Toolkit (Song et al., 2011), according to the pipeline from a previous study (Lin et al., 2014). (1) The first three scans were discarded due to magnetic saturation effects. (2) The imaging data were corrected for slice-timing order, realigned for head movement, and normalized to the Montreal Neurological Institute (MNI) template. (3) The time series was detrended and bandpass filtered (0.01–0.08 Hz) to extract the low-frequency oscillating components contributing to intrinsic FC. (4) Spurious or non-specific effects were removed by a regression model, with the following covariates: (a) the six movement parameters obtained from the image realignment procedure; (b) the mean signal within the lateral ventricles; and (c) the mean signal within the deep white matter. We did not perform a regression for the whole-brain global signal due to the debate on its effect (Murphy et al., 2009). The processed imaging data were subsequently used for the network analysis.

### Analysis of the Effect of Head Motion

Because head motion can significantly influence the results of the network analysis (Van Dijk et al., 2012), we compared the amount of head motion between the OA and the YA groups during the rs-fMRI scan. The motion parameters were extracted from the image realignment of the rs-fMRI data. For each participant, a head motion index was quantified as the mean displacement (i.e., the translation/rotation in the i^th^ time point relative to the (i − 1)^th^ time point), based on the method reported in a previous study (Liu et al., 2008). The head motion index was calculated for both translational motion and rotational motion. Between-group differences in the translational motion and rotational motion were examined using a two-tailed two-sample *t* test. We find a significant between-group (OA vs. YA) difference in the translation motion (OA > YA, two-tailed two-sample *t* test, *p* = 0.017) but no rotational motion (two-tailed two-sample *t* test, *p* = 0.38) during all scans. All participants showed a maximal displacement of <1.5 mm in translation and <1.5° in rotation.

### Graph-Based Network Analysis

**Figure 1:**
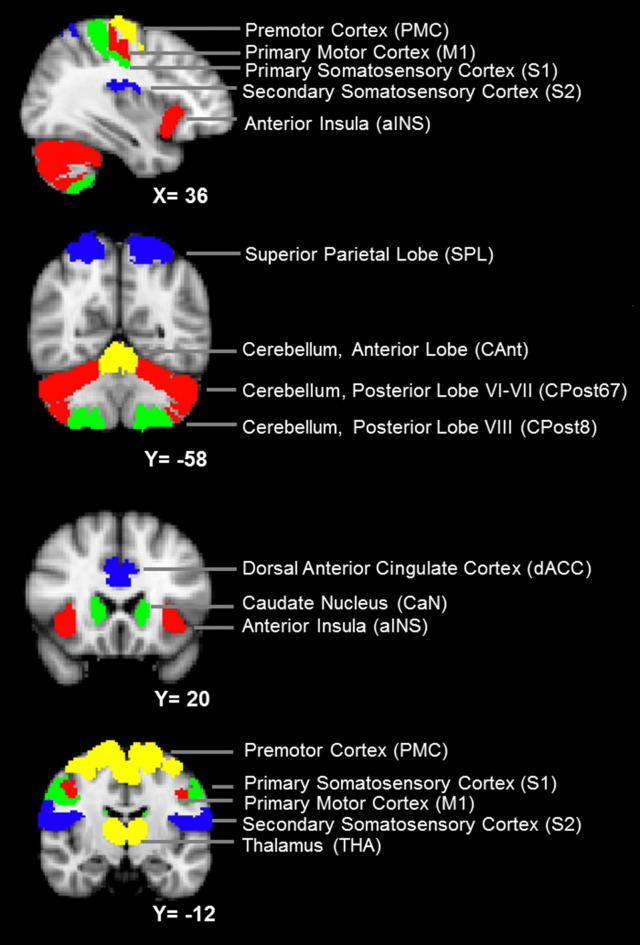
**The nodes of the mastication-related network.** See Table 1 for a detailed definition of the regions of interest (ROIs) of the nodes.

(a) Definition of the nodes: we assigned the nodes as the 12 brain ROIs related to motor control or mastication, based on a review of the literature (Figure 1 and Table 1). In addition to the primary motor cortex (M1), we included the secondary motor cortex, i.e., the PMC (including the supplementary motor area) and the dorsal anterior cingulate cortex (dACC, being part of the cingulate motor area; Paus, 2001). We included the subcortical regions, i.e., the thalamus (THA) and the caudate nucleus (CaN), due to their activation related to mastication in OA (Onozuka et al., 2003). We also included brain regions associated with sensory and cognitive processing, including the superior parietal lobe (SPL), the anterior insula (aINS) and the primary and secondary somatosensory cortices (S1/S2), due to their roles in sensorimotor integration and attention control in OA (Ward, 2006; Seidler et al., 2010). Finally, we included three sub-regions of the cerebellum: the anterior lobe (CAnt), lobule VI/VII of the posterior lobe (CPost67) and lobule VIII of the posterior lobe (CPost8), due to their connectivity with the cortical regions. The anterior and posterior sub-divisions contribute to sensorimotor and cognitive-affective processing, respectively (Stoodley and Schmahmann, 2010; Stoodley et al., 2012).
(b) Definition of the edges: we defined the edges of the FC network as the inter-nodal strength of the association of the time series, according to the following procedure. (1) We extracted the time series from all voxels within each node and calculated the mean regional time series. (2) For each pair of nodes, we calculated the Pearson correlation coefficient *(r)* between their regional mean time series. (3) The *r* value was converted to a *z* score using Fisher’s *r*-to-*z* transformation to improve the normality, followed by transformation to its absolute value. The absolute value of the *z* score was indexed as the weight of the network edge. Based on this procedure, an undirected and weighted network composed of 24 nodes and 276 edges was obtained for each participant.

**Table 1:** **Definition of the brain regions of interest (ROIs)**.

ROI	Abbreviation	Source	Thresholding
Primary motor cortex	M1	Jülich histological atlas, area 4a and 4p	0.5
Premotor cortex	PMC	Jülich histological atlas, area 6	0.5
Primary somatosensory cortex	S1	Jülich histological atlas area 1, 2 and 3	0.5
Secondary somatosensory cortex	S2	Manual definition, based on Wiech et al. (2014)	
Superior parietal lobe	SPL	Jülich histological atlas, area 7a	0.5
Anterior insula	aINS	Manual definition, based on Wiech et al. (2014)	
Dorsal anterior cingulate cortex	dACC	Manual definition, based on Wiech et al. (2014)	
Caudate nucleus	CaN	Harvard-Oxford subcortical anatomy atlas	0.5
Thalamus	THA	Harvard-Oxford subcortical anatomy atlas	0.5
Cerebellum, anterior lobe	CAnt	Probabilistic atlas of the human cerebellum, lobule I–V	0.5
Cerebellum, posterior lobe	CPost67	Probabilistic atlas of the human cerebellum, lobule VI, VIIb, Crus 1 and Crus 2	0.5
Cerebellum, posterior lobe	CPost8	Probabilistic atlas of the human cerebellum, lobule VIIIa and VIIIb	0.5

### Definition of the ROIs

The ROI of a node was designed according to anatomical probabilistic atlases or manually defined based on an independent group of subjects (Table 1 and Figure 1). M1, PMC, S1, SPL were defined according to the Jülich Histological Atlas (Eickhoff et al., 2007). CaN and THA were defined according to the Harvard-Oxford Subcortical Structural Atlas (Desikan et al., 2006). The cerebellum lobes (CAnt, CPost67 and CPost8) were defined according to the Probabilistic Cerebellar Atlas (Diedrichsen et al., 2009). These nine ROIs were designed, using FSLView^1^, by thresholding the probabilistic map with a group threshold of 0.5 (Table 1). S2, aINS and dACC were defined manually, according to the methods published in a previous study (Wiech et al., 2014; Table 1). The bilateral ROIs of a node were independently defined, and, therefore, 24 nodes were included in the network.

### Quantifying Network Degree Centrality

Our main hypothesis focused on the age-related difference in the mastication-related network. We calculated the graph index DC to quantify the importance of a node in the network. DC has been widely used to examine the local centrality of a node (Zuo et al., 2012). A higher DC value indicates that the node is highly connected and is a “hub” in the network. DC was calculated for each node by summing up the edge weights between this node and all of the other nodes.

### Statistical Analyses

(a) We identified the mastication-related hub, i.e., the node for which the DC was significantly positively correlated with the MPI. Partial correlation analyses were performed to investigate the correlation between the MPI and DC for each node, controlling for age and gender, across all participants in each age subgroup.
(b) For each node of the mastication-related network, we screened for the other nodes (*n* = 23) for which the FC with the target node was significantly correlated with the MPI, controlling for age and gender. Due to the exploratory nature of the analysis, we did not adjust the alpha value for multiple testing.

## Results

### Demographic and Clinical Profiles of the Study Group

**Figure 2:**
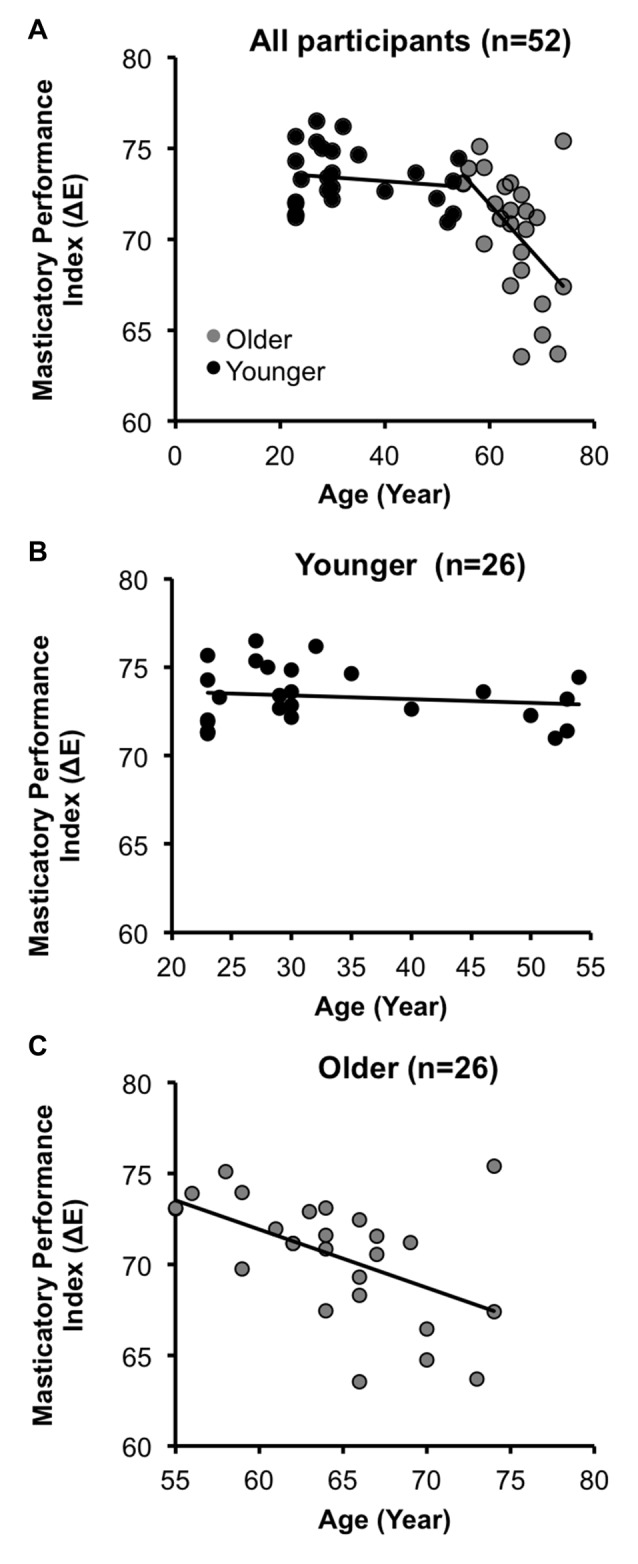
**Age-related decline in masticatory performance. (A)** Across all participants (*N* = 52), the masticatory performance index (MPI) decreased with increasing age. **(B)** In the younger adult group (YA), the correlation between the MPI and age was insignificant. **(C)** In the older adult group (OA), the MPI was significantly negatively correlated with age.

The demographic and clinical data are shown in Table 2. The mean MPI in the OA group was significantly lower than that in the YA group (OA: 70.5 ± 3.3, YA: 73.3 ± 1.6, two-tailed two-sample *t*-test *p* < 0.001). There was no significant gender difference in the number of subjects (*χ*^2^_(1)_ = 1.33, two-tailed *p* = 0.25). Across all participants (*n* = 52), age was significantly negatively correlated with the MPI (Pearson’s correlation coefficient *r* = −0.54, two-tailed *p* < 0.001). Specifically, within the OA group (*n* = 26), age was significantly negatively correlated with the MPI (*r* = −0.53, two-tailed *p* = 0.006), consistent with previous findings (Ikebe et al., 2011; Figure 2). In contrast, within the YA group, the correlation between age and the MPI was not statistically significant (Figure 2). The findings revealed that masticatory performance did not decline gradually as age increased. It showed a pattern of late-life decrease, i.e., starting to decline significantly during older age but not during younger age.

**Table 2:** **Demographic and clinical profiles**.

	Gender	Age		MPI	
		Mean ± SD	Max−Min	Mean ± SD	Max−Min
Total (*N* = 52)	19 (M)/33 (F)	48.7 ± 18.0	74–23	71.9 ± 3.0	76.5–63.5
Older (*N* = 26)	7 (M)/19 (F)	64.4 ± 5.4	74–55	70.5 ± 3.3	75.4–63.5
Younger (*N* = 26)	12 (M)/14 (F)	33.1 ± 11.1	54–23	73.3 ± 1.6	76.5–71.0
Older vs. Younger comparison^1^	*P* = 0.25	*P* < 0.001		*P* < 0.001	

*MPI, Masticatory performance index; SD, standard deviation. ^1^Comparison of the number of male and female subjects was performed by a chi-square test with Yate’s correction. Comparison of the subjects’ age and MPI was performed by a two-tailed independent *t*-test*.

### Identification of the Mastication-Related Hub

In the YA subgroup, we found the following mastication-related hubs, i.e., the nodes that showed a positive correlation between DC and the MPI: the left CAnt (*r* = 0.42, *p* = 0.032) and right CPost8 (*r* = 0.42, *p* = 0.034). In the OA subgroup, we found the following mastication-related hubs: the left PMC (*r* = 0.39, *p* = 0.048), left S2 (*r* = 0.41, *p* = 0.037), right S2 (*r* = 0.45, *p* = 0.021), right SPL (*r* = 0.48, *p* = 0.14), right aINS (*r* = 0.43, *p* = 0.027), left dACC (*r* = 0.45, *p* = 0.021) and right dACC (*r* = 0.42, *p* = 0.034).

### Network Analysis

**Figure 3:**
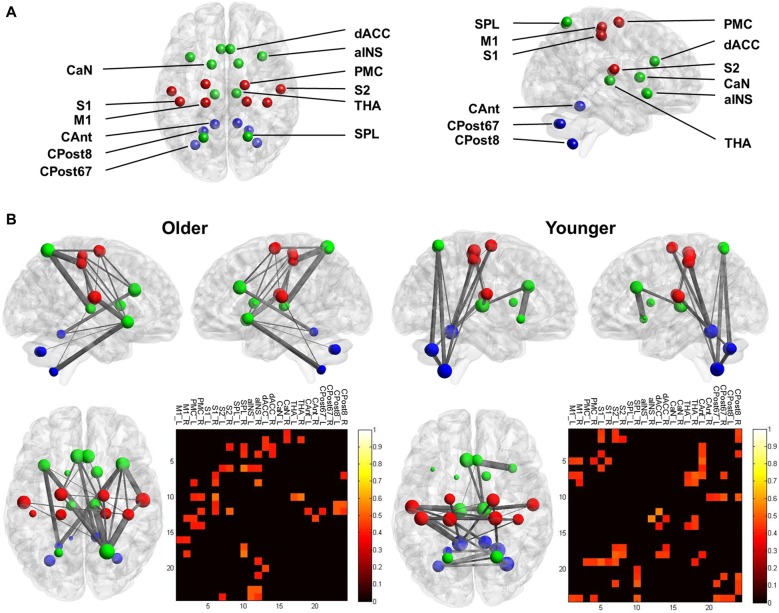
**Functional connectivity (FC) of the mastication-related network. (A)** The nodes that comprised the network. For visualization, the sensorimotor nodes (S1, PMC, S1 and S2) are colored red. The cerebellum nodes (CAnt, CPost67 and CPost8) are colored blue. All other cortical and subcortical nodes are colored green. See Table 1 for the abbreviation of each node. **(B)** The mastication-related FC network. The line between each node represents the FC that is statistically significant. Line thickness denotes the strength of association between the FC and the MPI. The pattern of the FC is displayed from the sagittal view (upper left and right) and the dorsal view (lower left) for both the younger and older groups. The lower right panel displays the matrix of FC between all pairs of nodes. The color denotes the strength of association between the FC and the MPI, quantified by Pearson’s correlation coefficient. Only the links with a significant correlation (*p* < 0.05) are displayed.

In the YA subgroup, we found a dense connection between the sensorimotor nodes (M1, PMC, S1 and S2), and a dense connection between the subcortical and cerebellum nodes. Notably, the cerebellum nodes showed a dense connection with the cortical sensorimotor nodes, including FC between the bilateral M1 and the right CPost8 and FC between the bilateral S1 and the left CAnt (Figure 3). In contrast, in the OA subgroup, the sensorimotor nodes were less connected (except for the bilateral S2). The cerebellum nodes also showed less connection. A dense connection was found between the sensorimotor nodes and the other cortical nodes, including the right SPL, the bilateral aINS and the dACC (Figure 3).

## Discussion

Previous studies have identified brain activation of the cerebellum and the motor cortex when participants were chewing (Onozuka et al., 2002; Quintero et al., 2013). In the current study, we further investigated the brain signatures associated with masticatory performance, a widely used index for evaluating the masticatory function of elderly people. In the YA group, the network analysis revealed that FC between the sensorimotor cortex, the THA and the cerebellum was positively correlated with the MPI (Figure 3). The findings suggested that younger people may automatically chew food without additional effort for monitoring or regulating their movement. Consistently, we found that the cerebellum, a key component for automatic control of movement (Ramnani, 2014), was identified as mastication-related hubs. In contrast, in the OA group, the network analysis revealed a sparser connection within the sensorimotor regions and cerebellum and a denser connection across distributed regions (Figure 3). Compared to the YA group, the OA group also comprised more mastication-related hubs, which were spatially distributed outside of the sensorimotor regions, including the right SPL, the right aINS, the left dACC and the right dACC. In general, the findings confirmed our main hypothesis that in OA, higher masticatory performance is associated with a more widespread pattern of mastication-related hubs compared to that observed in YA.

It has been widely accepted that motor function declines with aging (Ward, 2006). As shown in our behavioral findings, the decreased masticatory performance was associated with the increasing age, especially during late life (Figure 2). Cumulating evidence has suggested that the CNS plays a key role in the age-related decline of motor control. When performing a simple motor task (e.g., finger tapping) or rhythmic movement (e.g., chewing), older people show a more widespread engagement of multiple brain regions compared to that in younger people. In addition to the activation of M1, older people show more co-activation in cortical and subcortical regions (Mattay et al., 2002; Onozuka et al., 2003; Heuninckx et al., 2005; Wu and Hallett, 2005; Zwergal et al., 2012; Windel et al., 2015). For example, when performing a simple flexion-extension movement, older subjects showed an increased activity in the premotor area, the rostral cingulate and the prefrontal cortex, suggesting an increased cognitive monitoring of motor performance (Heuninckx et al., 2005). When performing paced finger movements, older subjects showed a greater extent of activation in the sensorimotor cortex, the premotor area (including the supplementary motor area), and the cerebellum (Mattay et al., 2002; Wu and Hallett, 2005). Importantly, research on human chewing, a movement maintained by CPG, also revealed an age-related difference in the activation pattern. Older subjects showed increased activity in the prefrontal area and decreased activity in the somatosensory cortex and the cerebellum (Onozuka et al., 2003). All of the findings suggest that in OA, motor control is associated with a widespread engagement of multiple brain regions.

According to the compensation theory, OA increasingly rely on cognitive processes for motor control (Seidler et al., 2010). The extended engagement of multiple brain regions may reflect the increased demand of multi-sensory integration, attentional control, or enhanced cognitive efforts for movement in OA (Ward, 2006; Seidler et al., 2010). Mastication is automatically maintained and rhythmically executed by the CPG of the brainstem (Avivi-Arber et al., 2011). Mastication can be automatically achieved without one’s overt attention. However, it also requires feedback and monitoring from the sensorimotor cortex. Our findings suggested that in OA, the FC between the sensorimotor cortex and other brain regions, rather than the FC within the sensorimotor cortex, shows a greater contribution to mastication. The extended engagement of the brain may indicate the distinct experience of chewing in older people. During mastication, OA need to monitor their oral status continually, perhaps due to poorer oral conditions (e.g., having fewer teeth to chew well). Consistently, we found a positive correlation between the MPI and bilateral PMC-right SPL connectivity, right PMC-aINS connectivity right S1-bilateral aINS connectivity and right S1-SPL connectivity (Figure 3). The aINS, as part of the salience network of resting-state FC, plays a predominant role in awareness of bodily perception (Menon and Uddin, 2010). The SPL plays a key role in maintaining an internal representation of the body’s state and contributes to sensorimotor integration (Wolpert et al., 1998). The finding may indicate that the OA need to overtly monitor the progress of mastication and intraoral perception (e.g., the size of food particles). This feedback information would be essential to maintain mastication.

Our findings echoed the recent neuroimaging evidence that revealed that the functional brain signature reflects an age-related difference. For example, increased age is associated with an increased long-range FC density (Tomasi and Volkow, 2012) and network efficiency (Song et al., 2014) in the sensorimotor network. The sensorimotor network also showed a pronounced decrease in segregation and increase in between-network connection (Chan et al., 2014). The age-related changes suggest that in OA, the ability to execute a motor task relies on the function of brain regions outside of the sensorimotor network, such as the SPL, the aINS and subcortical regions. The engagement of these regions may reflect increased feedback or “compensation” to facilitate the motor task (Ward, 2006; Seidler et al., 2010). One potential explanation of the age-related FC changes is brain plasticity, i.e., “an intrinsic property of the nervous system retained throughout a lifespan” (Pascual-Leone et al., 2005) that can be molded by long-term experience, such as motor exercise and sensory experience. Accumulating evidence has shown that changes in the FC network reflect changes in motor or sensory experience (Kelly and Castellanos, 2014). For example, changes in the connectivity of the SPL and the PMC were found in subjects who received long-term training in a coordination task, which demands integration of perceptual, motor and cognitive abilities (Demirakca et al., 2016). Compared to training of motor tasks, mastication is a more ordinary and common exercise for our daily life, and, therefore, alterations in masticatory function may reshape the related brain signatures.

We considered the following limitations crucial for interpreting the current findings. First, our conclusion is limited to the representativeness of the study group, which included all physically healthy OA with generally good oral health. We purposely excluded OA with motor or cognitive deficits, which may confound the results of the masticatory performance assessment or neuroimaging. The trade-off is that our conclusion may not reflect the broader spectrum of clinical cases in which people with extremely poor masticatory functions are commonly seen. Second, we did not make a direct and quantitative comparison of the brain connectivity between the YA and the OA groups. Instead, all of the investigation on FC was performed within the subgroups. We avoided a direct between-age subgroup comparison for two reasons: (a) there exists a significant age-related difference in brain anatomy (Good et al., 2001); and (b) our findings showed a significant difference in head translational motion between the age subgroups. These factors may confound the results of a direct comparison in FC between the age subgroups. Third, in the experimental design, we did not aim to clarify the causal link between the pattern of brain connectivity and masticatory performance. The positive correlation between DC and the MPI may indicate that individuals with such a brain organization can better adopt themselves to chew well. Conversely, it may indicate that the individuals who tried to chew well would gain more experience in chewing, resulting in re-organization of brain connectivity (i.e., an effect of brain plasticity). To clarify the causal link would require a further longitudinal investigation. Finally, though we focused on the cortical, subcortical and cerebellar changes related to mastication, it should not be ignored that mastication is predominantly maintained by the CPG of the brainstem. We did not directly assess the activity of the CPG due to the difficulty in precisely recording the signal from the brainstem (Brooks et al., 2013). Therefore, our results are limited to the pattern of cortical, subcortical and cerebellar signatures related to mastication.

Clinically, declined masticatory performance is related to worsening peripheral conditions, such as a decreased number of teeth, decreased saliva flow rate, or the reduction in biting force or masticatory muscle volume (Hatch et al., 2001; Ikebe et al., 2011). Our findings highlighted that, in addition to these peripheral factors, individual differences in the brain signatures may contribute to the age-related decline in masticatory performance. Decreased abilities in sensorimotor integration, attentional control or attentional monitoring, which are commonly age-related (Hedden and Gabrieli, 2004), may also contribute to the decline in the masticatory performance in OA. The findings suggest that, in addition to maintaining good oral health or installing a suitable dental prosthesis, training and exercise to increase oral motor and sensory functions play an important role in mastication. As age increases, a widespread engagement of cortical, subcortical and cerebellar regions may reflect an increased demand in sensorimotor integration, attentional control and monitoring for OA to chew.

## Author Contributions

CL initiated, conceived, performed the study, analyzed data and drafted the manuscript. CW, SW, DC and WL conceived the study and performed the study. H-HL performed the study. All authors have participated in discussion and writing of the final manuscript.

## Conflict of Interest Statement

The authors declare that the research was conducted in the absence of any commercial or financial relationships that could be construed as a potential conflict of interest. The reviewers and handling Editor declared their shared affiliation, and the handling Editor states that the process nevertheless met the standards of a fair and objective review.

## Footnotes

^1^https://fsl.fmrib.ox.ac.uk/fsl/fslwiki/FslView
